# Case Report: Rare aplastic anemia in pediatric systemic lupus erythematosus: a case series and systematic literature review

**DOI:** 10.3389/fped.2025.1602651

**Published:** 2025-06-27

**Authors:** Xianfei Gao, Ying Li, Xuerong Lan, Ping Zeng

**Affiliations:** Department of Pediatric, Allergy Immune and Rheumatology, Guangzhou Women and Children’s Medical Center, Guangzhou Medical University, Guangdong Provincial Clinical Research Center for Child Health, Guangzhou, China

**Keywords:** systemic lupus erythematosus, SLE, aplastic anaemia, AA, pediatric

## Abstract

**Background:**

Systemic lupus erythematosus (SLE) is a complex autoimmune disease in which the immune system mistakenly attacks healthy cells and tissues. This condition can impact various organs and systems, including the hematological system, where aplastic anemia (AA) emerges as a particularly severe and rare complication. Relevant clinical manifestations and treatment experiences must be shared and updated urgently.

**Methods:**

This manuscript presents the clinical features, examination, and treatment of two pediatric SLE patients with AA. And a systemic search of MEDLINE, EMBASE, LILACS, SciELO, and Scopus, using MeSH headings and keywords for “Aplastic Anaemia” and “Systemic Lupus Erythematosus”.

**Results:**

Both of our cases were severe, resulting in one death. Our systematic literature review identified 32 eligible studies. We found a total of 38 patients, and along with the two patients from our case series, there were 9 out of 40 SLE patients who experienced alopecia areata as children. In all 40 patients, 52% (21 out of 40) developed AA at the onset of SLE. The infection rate was concerning, with 16 out of 40 patients affected. While 83% (33 out of 40) of the patients showed improved outcomes, all seven patients who died had contracted an infection.

**Conclusion:**

SLE with AA is rare in both children and adults. Although the prognosis of this dangerous disease is optimistic in terms of data, it is still necessary to be vigilant, and early bone marrow examination, infection prevention, and on-time treatment are crucial.

## Introduction

Aplastic anemia (AA) is a rare but serious condition characterized by the bone marrow's inability to produce enough blood cells. This leads to a deficiency in the production of red blood cells, white blood cells, and platelets. AA can be a complication of systemic lupus erythematosus (SLE), an autoimmune disease characterized by widespread inflammation and tissue damage across various organs (1). The development of aplastic anemia in patients with systemic lupus erythematosus involves a complex interplay of immunological, genetic, and environmental factors. Although AA has a low incidence, its severe implications in SLE patients necessitate heightened clinical awareness and careful management (2). AA presents with a range of non-specific symptoms that may overlap with other hematological and systemic disorders. Common symptoms include fatigue and weakness due to anemia, bleeding tendencies from thrombocytopenia, increased susceptibility to infections from neutropenia, fever, pallor, and dyspnea. It is important to note that Macrophage Activation Syndrome can cause similar cytopenias but is typically associated with elevated ferritin levels, hemophagocytosis in the bone marrow, and increased transaminases. Infections may result in either leukocytosis or leukopenia; however, inflammation markers like CRP (C-reactive protein) or ESR (erythrocyte sedimentation rate) are usually elevated, and blood cultures may be positive in cases of bacteremia or septicemia. Drug-induced cytopenias may resemble AA but can show signs such as eosinophilia or increased blast cells without significant marrow hypoplasia. In severe cases, macrocytic anemia with pancytopenia may be observed, along with hypersegmented neutrophils and macro-ovalocytes on blood smears and deficiencies in Vitamin B12 and folate levels. Diagnosing AA in the context of SLE is complex due to the overlapping symptoms and varying complications. There is a need for more case reports that illustrate clinical manifestations and treatment experiences to enhance understanding of aplastic anemia in SLE and to develop appropriate treatment strategies (3).

In this article, we present a case series of two pediatric patients with AA as a complication of SLE, accompanied by a literature review discussing the clinical manifestations, diagnosis, and treatment of SLE complicated by AA.

## Case series

### Case 1

A 9-year-old Chinese girl was admitted to our hospital with a cough and malar rash for 1 month, and fatigue and hair loss for 10 days. Her paternal grandmother had skin cancer, and her maternal grandmother suffered from rheumatic heart disease. At time of admission, dark brown pigmentation was discovered around the fingernails on both sides. However, she had no history of trauma, other illnesses, or exposure to pigments. Investigation revealed an HB (hemoglobin concentration) of 86 g/L, WBC (white blood cell) 1.3 × 10^9^/L, Neutropenia 0.28 × 10^9^/L, PLT (platelet) 16 × 10^9^/L, ARC (absolute reticulocyte count) 0.006 × 10^12^/L, C3 (complement 3) 0.56 g/L, antinuclear antibody 1/80, urine RBC (red blood cell) 198/μl, 24-hour Urine protein 1.06 g/L, fibrinogen 4.86 g/L, ferritin 516.40 ng/ml, and CRP 38.3 mg/L. The patient suffered an Upper respiratory tract infection with Haemophilus influenzae in sputum culture and a normal chest CT. The patient was initially treated with MTX (methotrexate) 10 mg/week, prednisone 30 mg/day, Mercaptopurine 50 mg/day, and Cefuroxime for the infection. Bone marrow smear showed hypocellular marrow, and bone marrow biopsy showed cellularity of 10%, supporting AA. Due to bone marrow suppression, the patient discontinued mercaptopurine and began receiving intravenous methylprednisolone (starting with 500 mg daily), intravenous immunoglobulin (IVIG), granulocyte-colony-stimulating factor (G-CSF), and switched from MTX to mycophenolate mofetil (MMF). However, the HB kept on dropping to 67 g/L, PLT dropping to 13 × 10^9^/L, and the patients suffering dizziness, petechiae, and bloody stool. RBC and PLT transfusions were conducted. On the 23rd day of admission, the patient improved with HB 93 g/L, WBC 5.1 × 10^9^/L, and PLT 34 × 10^9^/L. The patient received regular intravenous methylprednisolone at a dosage of 5 mg/kg for 3 days each month, along with cyclophosphamide administered at 5 mg/kg per day for 2 days each month, over a period of 7 months. Following this, the same dosages were given every 2 months for an additional 4 months. After this treatment, the patient discontinued intravenous methylprednisolone and cyclophosphamide because the SLEDAI (Systemic Lupus Erythematosus Disease Activity Index) score had reduced to 5, and there was a high frequency of infections. The patient was maintained on oral steroids (2 mg/day), hydroxychloroquine (6.5 mg/kg/day), and MMF (400 mg/kg/day). After 5 years, the patient maintained SLEDAI scores of less than four.

### Case 2

A 9-year-old Chinese girl was admitted to our hospital with a half-month history of arthralgia in bilateral wrist and knee joints, a malar rash, and repeated fever (38.8℃) for 10 days. On evaluation she was noted to have oral ulcers, and labs showed an HB 83 g/L, WBC 2.7 × 10^9^/L, PLT 62 × 10^9^/L, C3 0.19 g/L, C4 (complement 4) 0.03 g/L, direct Coombs test 3+, antinuclear antibody titer of 1/640, anti-dsDNA (Anti-double-stranded DNA) antibody 1+. The SLE diagnosis was made, and the patient was initially treated with intravenous methylprednisolone and IVIG for 1 week, then MMF (0.75 g/day), regularly reduced oral steroids, and intravenous Benlysta (10 mg/kg) administered twelve times for 2 years. During this period, SLEDAI scored 2–5 points with low complement concentration, high anti-ds-DNA, or low platelet. However, at the age of 12, the patient took oral medications irregularly and did not have follow-up visits on time for about 1 year. Consequently, she experienced malar rash, pale lips, swelling of both eyelids, pitting edema of the limbs, chest pain, dizziness, fatigue, cough, and decreased urine output. The patient was hospitalized, and her evaluation revealed severe thrombocytopenia (54 × 10^9^/L) and anemia (57 g/L). She had low levels of C3 (0.15 g/L), C4 (0.02 g/L), a direct Coombs test reading of 3+, and low albumin (29.1 g/L). The infectious workup, which included tests for Epstein–Barr Virus (EBV), cytomegalovirus (CMV), Human Immunodeficiency Virus (HIV), Hepatitis C Virus (HCV), Hepatitis B Virus (HBV), and blood cultures, returned negative results. On the first day of her visit, a bone marrow smear showed active proliferation of granulocytes and erythroid cells, with visible megakaryocytes. Cardiac ultrasound revealed moderate pericardial effusion, while a chest CT scan indicated pulmonary congestion, bilateral pleural effusion, a small amount of condensed inflammation in both lungs, an enlarged heart shadow, and pericardial effusion. The patient was started on intravenous methylprednisolone, IVIG, and intravenous human albumin. Due to renal failure (BUN of 32.46 mmol/L) and high uric acid levels (753 µmol/L), she underwent plasma exchange and hemodialysis. For the elevated uric acid, she was treated with azathioprine. Subsequently, her ANC decreased to 0.06 × 10^9^/L and ARC to 0.003 × 10^12^/L, requiring RBC transfusions for critically low hemoglobin levels of 44 g/L and PLT transfusions for critically low levels of 2 × 10^9^/L. Due to a lack of response, a second peripheral blood and bone marrow smear performed on the 27th day revealed AA with hypocellular marrow. A bone marrow biopsy was not performed as the patient had low platelet counts and was at risk of bleeding. Her ferritin level was 1,590.30 ng/ml, while transaminases, fibrinogen, ESR, and CRP were normal. Macrophage activation syndrome (MAS) was excluded. The patient then developed Trichosporon cutaneum, confirmed by blood culture, and was treated with meropenem, linezolid, and caspofungin. On the 31st day, her myelosuppression improved with PLT levels rising to 75 × 10^9^/L, hemoglobin to 75 g/L, and WBC count to 10.2 × 10^9^/L. However, she began experiencing seizures due to intracranial hemorrhage. As her generalized edema worsened, along with refractory electrolyte imbalances and hypertension, she suffered another seizure, experienced loss of consciousness, and developed multiple intracranial hemorrhagic lesions. Ultimately, she passed away due to multiple organ failure.

### Systemic review of the literature

We performed a systemic search in several scientific databases (MEDLINE, EMBASE, LILACS, SciELO, and Scopus) using the following keywords: systemic lupus erythematosus, SLE, lupus, LES, aplastic anemia and aplastic anemia. We used MeSH-controlled vocabulary to index articles for MEDLINE. The study period ran from January 1950 through October 2024. Two authors independently screened all citations and abstracts to identify eligible studies. In addition, secondary references were obtained from the selected article. The inclusion criteria were English-language written and human articles. Both pediatric and adult SLE were included. Exclusion criteria were conference proceedings, abstracts, or editorials. Patients less than or equal to 18 years of age are defined as children, and adults are defined as older than 19.

## Results

Based on the data collected from 32 publications and our presented cases, there are 40 cases of SLE with AA. Females were the dominant gender, with 35 patients. Among the 19 patients who developed AA after SLE, the duration from the onset age of SLE to AA varies from 0.5 to 22 years, as shown in Table 1. Children (patients aged ≤18 years) occupied about 9 (23%) of this rare condition. There was no noticeable difference among different races numerically.

**Table 1:** Summary of published studies regarding SLE with aplastic anemia in pediatric patients, associated factors, and outcome.

N	Sex	Age at SLE diagnosis (year)	Age at AA diagnosis (year)	Duration from onset age of SLE to AA (year)	Race	Type of study/population	Neutropenia account (/μl) and duration	Infections during AA	Pathology	Azathioprine before AA	Outcome
1	F	9	9	0	Asian (Chinese)	Our case report/2	280	Y	Haemophilus influenza	Y	Recovery
2	F	9	12	3	Asian (Chinese)		60	Y	Trichosporon cutaneum	Y	Dead
3	F	67	67	0	Asian	Case report/2 (13)	820	N	N	N	Recovery
4	F	70	70	0	Black (Indian)	Case report/2 (13)	660	N	N	N	Recovery
5	F	22	23	1	Black	Case report/1 (3)	1,800	Y	Epstein–Barr Virus	N	Recovery
6	F	26	26	0	Unknown	Case report/1 (24)	1,200	N	N	N	Recovery
7	F	31	31	0	White	Case report/1 (25)	Unknown (WBC 1,900),	N	N	N	Recovery
8	F	22	25	3	Unknown	Case report/1 (26)	Unknown (WBC 3,000),	N	N	Y	Recovery
9	M	28	28	0	Unknown	Case report/1 (27)	Unknown (WBC 1,970)	Y	HBV	N	Recovery
10	F	31	53	22	Asian (Vietnamese)	Case report/1 (1)	Unknown (WBC 700)	Y	Methicillin-sensitive staphylococcus aureus, Candida tropicalis, Bacteroides thetaiotaomicron, Rhizopus fungi	Y	Dead
11	F	29	29	0	Unknown	Case report/1 (14)	Unknown (WBC 1,300)	N	N	N	Recovery
12	F	52	52	0	Unknown	Case report/1 (2)	1,442	N	N	N	Recovery
13	F	41	42	1	Black	Case report/1 (28)	190	Y	Candida albicans	N	Dead
14	F	22	23	1	Black	Multicenter study/1 (29)	0	N	N	N	Recovery
15	F	6	6	0	Unknown	Case report/1 (30)	Unknown (WBC 1,980)	N	N	N	Recovery
16	F	25	25	0	Unknown	Case report/1 (31)	Unknown (WBC 900)	N	N	N	Recovery
17	F	39	39	0	Unknown	Case report/1 (32)	580	Y	HIV, Pneumococcal meningitis	N	Recovery
18	F	54	54	0	Unknown	Case report/1 (33)	112	N	N	N	Recovery
19	F	22	22	0	Unknown	Case report/1 (34)	845	N	N	N	Recovery
20	F	42	56	14	Unknown	Case report/1 (35)	625	Y	Staphylococcus aureus Pseudomonas aeruginosa	Y	Dead
21	F	32	32	0	Unknown	Case report/1 (36)	299	Y	Sepsis, aspiration pneumonia	N	Dead
22	M	26	26	0	Unknown	Case report/2 (37)	260	Y	Staphylococcus aureus	N	Recovery
23	F	23.5	24	0.5	Unknown		0	N	N	Y	Recovery
24	F	26	29	3	Asian (Japanese)	Case report/1 (38)	<500	Y	Brain abscess	N	Dead
25	M	33	33	0	White (Hispanic)	Case report/2 (39)	Unknown (WBC 2,800)	N	N	N	Recovery
26	F	35	35	0	Unknown		Unknown (WBC 400)	N	N	N	Recovery
27	F	36	41	5	Unknown	Case report/1 (40)	Unknown	N	N	N	Recovery
28	F	3	6	3	Unknown	Case report/1 (22)	0	N	N	N	Recovery
29	F	17	18	1	White	Case report/1 (41)	192	N	N	Y	Recovery
30	F	48	48	0	White	Case report/1 (42)	918	N	N	N	Recovery
31	F	42	44	2	Unknown	Case report/1 (43)	480	Y	Staphylococcus aureus	Y	Recovery
32	M	19	27	8	Black	Case report/2 (44)	465	N	N	N	Recovery
33	F	25	30	5	Black		215	Y	Staphylococcus aureus	N	Recovery
34	F	36	36	0	Black	Case report/1 (45)	500	N	N	N	Recovery
35	F	13	14	1	White	Case report 1 (46)	180	N	N	N	Recovery
36	F	17	17.5	0.5	Unknown	Case report 1 (47)	40	Y	Staphylococcus aureus	N	Recovery
37	F	16	16	0	White	Case report 2 (48)	1,340	N	N	N	Recovery
38	M	15	15	0	White		1,310	N	N	N	Recovery
39	F	14	27	13	Unknown	Case report 2 (16)	48	Y	Unknown	Y	Dead
40	F	42	54	12	Unknown		839	Y	Unknown	Y	Recovery
Total	F 35/M 5	C(10)/A(30)	C(9)/A(31)		Black 7/Asian 5/White 7/unknown 21	No. of Publications 32 (40 cases)		Y 16/N 24		Y 10/N 30	Recovery33 (83%)

C, child; A, adult.

Infections occurred in 16 of 40 patients (40%), with Staphylococcus aureus as the most frequent pathogen. The survival rate for infected patients (9 of 16 with infection = 56%) was lower than the overall survival rate (33 of 40 overall = 83%). Both pediatric patients from our case series had an azathioprine history. A summary and the literature review results show that 10 patients with an azathioprine history were found before AA onset (Table 1). The outcome for SLE with AA was promising, with a survival rate of 83%. Notably, all 7 cases with fatal outcomes (Patients No. 8, 11, 18, 19, 22, 37, 39) had infections during AA treatment.

The treatments are shown in Table 2. Corticosteroids were the primary medication in the treatment, administered to 95%, followed by blood transfusion at 55%, cyclosporine at 30%, HCQ at 28%, IVIg at 25%, etc. Only seven patients (18%) received standard SAA treatments. Standard SAA treatment was immunosuppressive therapy (IST) with ATG and cyclosporine, or hematopoietic stem cell transplant (4).

**Table 2:** Treatment of SLE with AA.

N	Corticosteroids	Cyclosporine	IV Ig	RTX	HCQ	ATG	Blood transfusion	G-CSF	Other therapies	Standard AA treatment
1	Y	N	Y	N	N	N	Y	N	Plasma exchange	N
2	Y	N	Y	N	N	N	Y	N	IV CTX Antibiotic MMF	N
3	Y	Y	N	N	N	Y	Y	N	IV CTX Eltrombopag	Y
4	Y	Y	Y	Y	N	Y	Y	N	Plasmapheresis Eculizumab	Y
5	Y	Y	Y	Y	Y	Y	Y	N	MMF IV CTX Fludarabine Total irradiation	Y
6	Y	N	N	N	N	N	N	N	N	N
7	Y	N	N	N	N	N	N	N	IV CTX. Antibiotic	N
8	Y	N	N	N	N	N	N	N	IV CTX.	N
9	Y	Y	Y	Y	Y	N	Y	N	Plasmapheresis IV CTX. MMF Tenofovir Eltrombopag MTX	N
10	Y	N	N	N	N	N	Y	Y	Amphotericin B, Posaconazole, Vancomycin, Meropenem	N
11	Y	Y	N	Y	Y	N	Y	N	Danazol IV CTX.	N
12	Y	Y	N	N	Y	N	N	N	MTX	N
13	Y	Y	N	N	N	Y	N	N	Oral dapsone Amphotericin B Antimicrobial Chemotherapy	Y
14	Y	Y	Y	Y	Y	Y	Y	Y	MMF Allogeneic Hematopoietic stem cell transplant	Y
15	Y	Y	N	N	N	N	N	N	N	N
16	Y	Y	Y	Y	N	Y	Y	Y	N	Y
17	Y	N	N	N	Y	N	Y	N	HAART	N
18	Y	N	N	N	N	N	N	N		N
19	Y	Y	N	N	N	N	Y	N	Phenytoin Phenobarbitone IV CTX.	N
20	Y	Y	N	N	N	Y	Y	Y	Imipenem/cilastatin Clindamycin Vancomycin Hydrochloride, SulfamethoxazoletrimEthoprim, Amphotericin B, and Fluconazole	Y
21	Y	N	N	N	N	N	N	N	N	N
22	Y	N	Y	N	Y	N	Y	N	Antibiotic Erythropoietin	N
23	Y	N	N	N	N	N	Y	N	Antibiotic Erythropoietin	N
24	Y	N	N	N	N	Y	Y	Y	N	N
25	N	N	N	N	N	N	N	N	Patinib	N
26	Y	N	N	N	N	N	N	N	N	N
27	Y	N	N	N	N	N	Y	N	N	N
28	Y	N	Y	N	N	N	N	N	N	N
29	Y	N	N	N	N	N	N	N	Plasma exchange	N
30	Y	N	N	N	N	N	N	N	Plasma exchange IV CTX	N
31	Y	N	N	N	N	N	N	N	Antibiotic Indoprofen Raniidine	N
32	Y	N	N	N	N	N	Y	N	Clindamycin Gentamcin	N
33	Y	N	N	N	N	N	Y	N	Plasma exchange Phenobarbital4Diphenylhydantion	N
34	Y	N	N	N	N	N	Y	N	Androgen therapy Plasma exchange	N
35	Y	N	N	N	Y	N	N	N	N	N
36	Y	N	N	N	N	N	Y	N	Plasmapheresis Antibiotic	N
37	N	N	N	N	Y	N	N	N	N	N
38	Y	N	N	N	Y	N	N	N	N	N
39	Y	N	Y	Y	N	N	N	N	MMF Tacrolimus IV CTX Antibiotic	N
40	Y	N	N	Y	Y	N	Y	N	MMF Antibiotic	N
Total	Y 38 (95%)	Y 12 (30%)	Y 10 (25%)	Y 8 (20%)	Y 11 (28%)	Y 8 (20%)	Y 22 (55%)	Y 5 (13%)		Y 7 (18%)

IV Ig, intravenous immunoglobulins; RTX, rituximab; HCQ, hydroxychloroquine; G-CSF, granulocyte colony-stimulating factor; ATG, antithymocyte globulin; IV, intravenous; CTX, cyclophosphamide; MMF, mycophenolate mofetil; MTX, methotrexate; HAART, tenofovir, lamivudine, ritonavir, and atazanavir.

## Discussion and conclusions

Acquired aplastic anemia is a rare, life-threatening hematological disorder characterized by bone marrow failure resulting in pancytopenia, or the reduction of red blood cells, white blood cells, and platelets. Even though the mechanism of acquired aplastic anemia is unclear, several factors have been identified as potential triggers. (1) Environmental factors. Exposure to certain toxins and chemicals, such as benzene and its derivatives, increases the risk of developing aplastic anemia (5). (2) Pharmacological Agents. Various medications have been associated with the onset of aplastic anemia. Notable culprits include nonsteroidal anti-inflammatory drugs (NSAIDs), antiepileptics, and certain antibiotics such as chloramphenicol. Chemotherapeutic agents, such as Azathioprine, and radiation have also been causative in some cases (6). (3) Viral Infections. Viral infections are another significant risk factor. Hepatitis viruses, Epstein–Barr Virus (EBV), Cytomegalovirus (CMV), and parvovirus B19 have been linked to aplastic anemia through mechanisms under investigation (7–10). (4) Immune System Dysregulation. The immune system's inappropriate attack on hematopoietic stem cells within the bone marrow is a critical mechanism in acquired aplastic anemia. SLE is one of the reasons. It involves autoreactive T-cells and the overproduction of cytokines like gamma-interferon and tumor necrosis factor-alpha (TNF-α), leading to hematopoietic suppression (11).

The revised Camitta criteria categorize AA into three classes based on BM cellularity, absolute neutrophil count (ANC), platelet count, and absolute reticulocyte count (ARC). Severe aplastic anemia (SAA) is identified by the presence of pancytopenia, with ANC below 0.5 × 10^9^/L, platelet count below 20 × 10^9^/L, and reticulocyte count below 20 × 10^9^/L as measured by manual count, or below 60 × 10^9^/L when using an automated analyzer. Additionally, diagnosis of AA also requires a hypocellular bone marrow with bone marrow cellularity of less than 25%, as measured by bone marrow core biopsy. Very severe aplastic anemia (vSAA) meets the same criteria, except for an ANC of less than 0.2 × 10^9^/L. Non-Severe Aplastic Anemia (NSAA) patients have depressed blood counts but do not meet the specific criteria for SAA or VSAA (12).

The overlapping clinical features of SLE with AA necessitate a high index of suspicion for diagnosis. Diagnosing AA in patients with SLE is crucial yet often challenging, leading to delays in diagnosis and negative outcomes. Most studies in this field consist mainly of case reports; therefore, multicenter research is necessary to support statistical and clinical analysis (13).

In our study, the first case presented both SLE and AA occurring simultaneously at the onset. The underlying pathophysiological connection arises from autoimmunity and the immune-mediated destruction of hematopoietic progenitor cells. This comorbidity complicates diagnosis and treatment, emphasizing the necessity for a nuanced understanding of effective clinical management (14). Although bone marrow biopsy is invasive, it should be considered when an SLE patient presents with blood system-related symptoms (3).

Managing therapeutic interventions for SLE in patients with concurrent AA poses challenges for physicians. Even though 83% of the patients from our literature review achieved remission, infection is a significant threat to SLE patients with AA, as all the patients with fatal outcomes suffered from infection. Overall survival for patients with infections was only 38%. Both of the cases reported here suffered from infections, and only one of them survived.

Regardless of severity, supportive care is fundamental in managing aplastic anemia. This includes Transfusions, Infection Prevention, and Routine Monitoring. For SAA and VSAA, the combination of ATG and CsA is the frontline treatment for patients who are not candidates for hematopoietic stem cell transplantation (HSCT). ATG targets and depletes T-cells, reducing their attack on bone marrow, while CsA inhibits T-cell activation and proliferation. Eltrombopag, a thrombopoietin receptor agonist, is sometimes included in the treatment regimen to stimulate bone marrow and increase platelet production. HSCT offers a potential cure for eligible patients, especially younger ones with a matched sibling donor. The procedure involves replacing the damaged bone marrow with healthy cells from a donor. Advances in transplantation techniques have improved outcomes significantly. Patients with NSAA typically exhibit a less aggressive disease course. Treatment may involve watchful waiting and symptomatic management (15).

However, for specific SLE-induced AA, the treatment strategy often focuses more on SLE (16). Corticosteroids are commonly used as the first-line treatment for SLE (17). Calcineurin inhibitors (CNI) like cyclosporine effectively reduce immune-mediated destruction in AA and control SLE activity, making them crucial for managing both conditions concurrently. Azathioprine is a cornerstone drug in managing SLE and exerts immunosuppressive effects by inhibiting purine synthesis, thereby reducing the proliferation of lymphocytes. Thus, azathioprine use should be approached with caution, particularly in patients presenting with unexplained cytopenias (18). Biologic agents targeting specific pathways in SLE are emerging as potential therapeutic options in this dual-diagnosis scenario (19). Rituximab, a monoclonal antibody targeting CD20-positive B cells, reduces autoantibody production and may mitigate some autoimmune mechanisms contributing to AA (14). Antithymocyte globulin (ATG), often used in severe AA cases, can reduce bone marrow suppression. Combining ATG with CNI has shown efficacy; however, its impact on SLE must be continuously monitored (20). Plasma exchange reduces the autoimmunity burden by clearing the pathological factors, potentially improving marrow function over time, and sometimes offers an optimum treatment option. For patients with both SLE and AA, allogeneic HSCT can provide a potential cure, but this approach carries high risks, including graft vs. host disease and the potential for severe autoimmune flares (21).

Supportive care is also crucial for patients with SLE and AA. Regular blood transfusions for anemia, platelet transfusions for thrombocytopenia, and granulocyte colony-stimulating factor (G-CSF) for neutropenia can be critical in managing acute, life-threatening events. Given these patients' immunocompromised state, prophylactic antibiotics are essential to prevent infections, which can have catastrophic consequences (22, 23).

In conclusion, cytopenias are commonly seen at the onset of SLE, and bone marrow aspirate and biopsy should be considered for patients with multiple lineages affected in order to rule out AA. AA should be regarded when there are changes in other systems involved in SLE. The outcomes of these patients were numerically optimistic, but infection poses a lethal threat, and prophylactic antibiotics should be considered.

## Data Availability

The original contributions presented in the study are included in the article/Supplementary Material, further inquiries can be directed to the corresponding author.
